# Reexamining the calculations of exercise energy expenditure in the energy availability equation of free-living athletes

**DOI:** 10.3389/fspor.2022.885631

**Published:** 2022-10-24

**Authors:** Motoko Taguchi, Melinda M. Manore

**Affiliations:** ^1^Faculty of Sport Sciences, Waseda University, Saitama, Japan; ^2^Nutrition Department, College of Public Health and Human Sciences, Oregon State University, Corvallis, OR, United States

**Keywords:** energy availability, non-exercise activity thermogenesis, exercise energy expenditure, athletes, doubly labeled water

## Introduction

For athletes, chronic energy deficiency termed low energy availability (EA) is a significant issue in both female and male athletes (1, 2). EA is defined as the amount of dietary energy remaining for other body functions after the energy cost of exercise is covered and normalized to fat-free mass (FFM) (or lean body mass) (3). The conventional EA equation is as follows:


$$\begin{array}{l} EA\left(kcal/kgFFM/day\right)=\left[EI\left(kcal/day\right)-EEE\left(kcal/day\right)\right]/FFM\;\left(kg\right) \end{array}$$

EI = energy intake; EEE = exercise energy expenditure.

An EA of < 30 kcal/kg FFM/day is typically defined as clinically low EA (4). Since the introduction of EA in 2007 (5), numerous researchers have assessed EA in athletes with equivocal results, due in part to no clear methodological guidelines for calculating EA, including techniques used to measure each component of the EA equation (6, 7). For example, female athletes with similar EI had different menstrual conditions (eumenorrheic or amenorrheic) (8, 9), while in males, EI is similar between cross-country athletes and sedentary controls (10). Conversely, the mean EEE in female and male athletes at risk for low EA was significantly higher than moderate or no-risk athletes (11, 12), suggesting that in athletes, high EEE affects EA values. Athletes participating in high levels of exercise do not appear to be eating adequately to cover EEE. This inconsistency may be attributed to the difficulty in accurately measuring EI and EEE. The assessment difficulties of EI are well documented (6), but the components to be included in EEE are less frequently examined. To date, only one study has attempted to calculate EA based on different methods for estimating EEE (8). Therefore, the goal of this opinion piece is to outline the rationale for including non-exercise activity thermogenesis (NEAT) as part of EEE in the EA equation to improve estimates of EA.

## Calculation of EEE in free-living athletes

Assessing of EEE in the field is challenging and typically only includes exercise expended in sport training. To demonstrate the difficulty in determining EEE, Guebels et al. (8) measured EA in female college athletes, with or without menstrual dysfunction, using different methods for quantifying EEE. They measured total energy expenditure (TEE) using 7-day activity logs, accelerometers, and running energy expenditure on the treadmill to assess more accurately “planned EEE” and then EEE calculated using four different methods. Method 1 comprised of all planned exercise that included exercise training and all purposeful physical activity (PA) regardless of intensity but did not include PA that resulted from social games, hobbies, leisure pastimes, or transport-related activity (< 30 consecutive min). Method 2 included all planned exercise plus bicycle commuting and all walking. This method also added transport-related activities as planned PA. For consistency, bicycle commuting was entered as general/leisure bicycling of 4.0 metabolic equivalent (METs) and all walking was entered as moderate-intensity walking (3.3 METs). No other activities were identified as being equal to 4.0 or 3.3 METs; walking (lasting for ≥30 consecutive min or within an exercise workout) was included in Method 1. Method 3 included all exercise at ≥4 METs. This method quantified EEE more objectively using a 4.0 MET cut-off, which incorporates the bicycle commutes but excluded walking of ≥3.3 METs. Method 4 included all exercises of >4 METs and included all the activities from Method 3, except for the bicycle commutes (4.0 METs). As expected, the more activities were included in EEE, the lower the EA value. This means that EA values varied widely depending on how EEE was qualified.

## Alternative method for calculating EEE in free-living athletes

TEE comprises four components: resting metabolic rate (RMR), diet-induced thermogenesis (DIT), NEAT, and EEE (13). Activity-induced energy expenditure (AEE) refers to the energy obtained by subtracting DIT and RMR from TEE (14), that is, the sum of planned exercise or sport exercise training and NEAT. Athletes often perform spontaneous exercises such as swimming and running, in addition to their scheduled training. Almost all previous EA studies have included only the energy expenditure of planned training as EEE and do not include PA performed in their daily lives. In addition, some athletes may spend more than an hour commuting to school/work by bicycle over the intensity of 4.0 METs. For endurance runners, the mean AEE was 1,688 kcal/day (47% of TEE) in males (15), and 1,585 kcal/day (52% of TEE) in females (16), accounting for approximately half of the TEE. Since energy used to support one process cannot be used for others (17), accurate measurement of EA depends on how accurately and realistically EEE is assessed. A method that includes NEAT and planned exercise in EEE is more suitable for free-living athletes than the conventional method. Reassessing how EEE is calculated will allow for more accurate predictions of EA and the ability to detect energy-deficient athletes earlier. Therefore, we propose that the EA calculation in free-living athletes should be as follows:

Improved EA (kcal/kg FFM/day) = [EI (kcal/day) – AEE (kcal/day)]/FFM (kg), where AEE includes programmed EEE and NEAT.

## Alternative methods for detecting low EA without measurement of EI or EEE

Early detection of athletes at risk of energy deficiency is essential, regardless of gender, age, or sports events. Owing to difficulties and errors in measuring EI, EEE and FFM, which are the components of EA, other potential surrogate markers for low EA have been investigated (7). The RMR ratio, measured RMR divided by predicted RMR, is an acceptable indicator of low EA regardless of race and sex (18–22). The “field method” would allow for identification of athletes at risk for low EA without assessing EI or EEE. To calculate this ratio, it is necessary to both measure and estimate the RMR. Thompson and Manore (23) showed that FFM should be used to calculate RMR estimates for athletes and that the Cunningham equation was the most suitable for RMR estimation in male and female athletes. The Cunningham equation is also widely used to estimate the RMR in White individuals (19, 24). The tissues and organs that are components of FFM are not energetically equal and have specific metabolic rates. Therefore, the dual-energy x-ray absorptiometry (DXA) equation, which is obtained by measuring body composition with high accuracy using DXA and multiplying it by the value of the RMR of each tissue, has been utilized (20, 24). Race was found to be a significant predictor of RMR after adjusting for age, sex, body mass index, fat mass, and FFM, and it is appropriate to use an RMR equation that matches the population's characteristics (22). In addition, there is a cut-off value suitable for each RMR estimation method to determine the RMR ratio (25).

In response to periods of low EA, the hypothalamic-pituitary-thyroid axis adapts to reduce energy expenditure (26). Athletes with menstrual disorders have demonstrated consistently decreased triiodothyronine (T_3_) levels (9, 27), therefore, a low T_3_ level is one objective blood marker that could be used to identify female athletes with low EA. In exercising men, it has been reported that leptin and insulin are reduced, independent of whether low EA had originally occurred with or without exercise; however, low EA did not significantly impact ghrelin, T_3_, testosterone, and insulin-like growth factor-1 (IGF-1) levels (28). Another study indicated a significant positive association between IGF-1 and the RMR ratio in highly-trained male soccer players (29). Further research regarding male athletes' endocrine adaptive processes to exercise training and response to reduced EA is necessary.

## Discussion

### Better method for EEE

**Figure 1:**
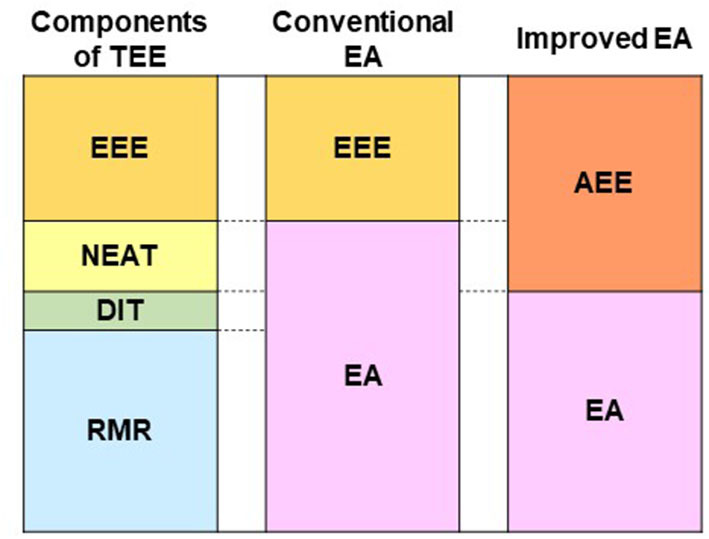
Components of TEE, components of conventional EA equation, and components of improved EA equation. TEE, total energy expenditure; EA, energy availability; RMR, resting metabolic rate; DIT, diet-induced thermogenesis; NEAT, non-exercise activity thermogenesis; EEE, exercise energy expenditure; AEE, activity-induced energy expenditure. Conventional EA (kcal/kg FFM/day) = [EI (kcal/day) – EEE (kcal/day)] / FFM (kg). Improved EA (kcal/kg FFM/day) = [EI (kcal/day) – AEE (kcal/day)] / FFM (kg).

For athletes, EA is the residual energy available to support physiological functions after covering the costs of physical activity. However, the EA equation and low cut-off value was derived in a metabolic laboratory-based study based on the impairment of hormones related to the female reproductive cycle in eumenorrheic, weight stable, and sedentary women (4, 6). This EA concept only accounts for EEE of planned exercise in the laboratory setting and NEAT was low. NEAT varies with environmental factors, activity status, physiological factors, and occupation, and can vary up to 2,000 kcal/day in individuals (30), even with similar body sizes (31). While the lack of consideration of NEAT in the calculation of EA outside the laboratory provides simplicity of EA calculation, it poses a potential “noise” factor for the comparison of EA between studies or for using universal EA threshold values (13). This may skew the true EA for physiological functionality in active populations (32). Therefore, we suggest an improved EA calculation that includes NEAT (Figure 1). NEAT should include the energy expenditure outside of planned sport training such as voluntary exercise training, strength training, cycling exercise using a bicycle ergometer, swimming, and biking to school/work. Methods available in the field to measure NEAT are accelerometer (29), multisensor armband (32), or calculation from activity logs using METs (8). If the doubly labeled water (DLW) technique is available, there is a laboratory method of calculating NEAT by subtracting RMR, DIT (0.1TEE), and EEE from TEE (33).

Lee et al. (29) reported that the EA of collegiate soccer players calculated by the conventional equation was 31.9 ± 9.8 kcal/kg FFM/day. A recalculation of EA for the same participants using the AEE approach resulted in an EA of 19.7 ± 8.5 kcal/kg FFM/day. The number of participants with LEA (< 30 kcal) increased from 5 to 10 using AEE instead of EEE with the improved equation. All five participants with newly classified LEA had lower testosterone levels, and higher bone resorption markers than the reference value. Thus, these participants would be considered at risk for future health issues caused by LEA, making early detection of at-risk athletes more realistic by improved EA equation.

### Better methods for EI

As mentioned earlier, EI is a critical component of EA and is known to be underestimated (6). DLW is the gold standard for measuring TEE under free-living conditions, and the TEE measured by DLW can be considered an EI if body weight is stable (34). To eliminate the underestimation of EI by participants in EA studies, research assessing EI using DLW could be used in the EA calculation. It is also necessary to measure body composition in relation to FFM with high accuracy using DXA. So far, only one study (35) has combined DLW and DXA to determine EA (kcal/day) of athletes. In this study, the EA at the beginning of the season was ~39.1 kcal/kg FFM/day in male athletes and 42.9 kcal/kg FFM/day in female athletes. These values are higher than those reported in previous studies of both sexes using EI values obtained from dietary records (36, 37). The Food Frequency Questionnaire (FFQ) is often used to calculate EI in EA studies because it is less burdensome and more cost-effective. However, the FFQ tends to overestimate EI in low-energy consumers and underestimate EI in large eaters (38); thus, researchers and dietitians should be careful in EA evaluation using FFQ.

Taken together, it is crucial to build evidence for the physiological effects of low EA by facilitating studies that can more accurately measure the components of EA, including using the DLW surrogate for EI, adding NEAT in EEE, and accurately measuring FFM. Better laboratory-based measurements will help researchers develop a more accurate, cheaper, and simpler field method for calculating EA. A better field method for EA will standardize and improve the identification of free-living athletes at risk for energy deficiency and associated health issues that occur if chronic energy deficiency persists. Furthermore, knowing an athlete's EA can help in developing diet plans that more accurately help an athlete meet their needs.

## Author contributions

MT and MM conceived the idea for this manuscript, developed the outline, and compiled the manuscript. Both authors contributed to the article and approved the submitted version.

## Conflict of interest

The authors declare that the research was conducted in the absence of any commercial or financial relationships that could be construed as a potential conflict of interest.

## Publisher's note

All claims expressed in this article are solely those of the authors and do not necessarily represent those of their affiliated organizations, or those of the publisher, the editors and the reviewers. Any product that may be evaluated in this article, or claim that may be made by its manufacturer, is not guaranteed or endorsed by the publisher.
